# Long-term application of fertilizer and manures affect P fractions in Mollisol

**DOI:** 10.1038/s41598-020-71448-2

**Published:** 2020-09-09

**Authors:** Xinchun Lu, Al-Kaisi Mahdi, Xiao-zeng Han, Xu Chen, Jun Yan, Asim Biswas, Wen-xiu Zou

**Affiliations:** 1 Northeast Institute of Geography and Agroecology, Chinese Academy of Sciences, 138 Haping Rd, Harbin, 150081 People’s Republic of China; 2 Department of Agronomy, Iowa State University, Ames, IA USA; 3 School of Environmental Sciences, University of Guelph, 50 Stone Road East, Guelph, ON N1G 2W1 Canada

**Keywords:** Agroecology, Environmental impact

## Abstract

Application of phosphorus (P), a major plant nutrient, as fertilizer is critical to maintain P level for crop production and yield in most cultivated soils. While, it may impact the dynamics, limited studies have examined the long-term effects of fertilization on P fractions in a soil profile in Mollisol. A long-term field experiment was conducted at the State Key Experimental Station of Agroecology of the Chinese Academy of Sciences in Hailun county, Heilongjiang Province, China. A sequential fractionation procedure was used to determine the effect of fertilizer (types) treatments including no fertilizer (CK), chemical fertilizer (NPK), chemical fertilizer plus straw (NPK + S) and pig manure (OM) on fractions of P and their distribution within 0–100 cm soil profiles. Unlike CK treatment, the long-term application of fertilizers increased the concentration and accumulation of total and available P in 0–20 and 0–40 cm soil depths than deeper soils, respectively. The phosphorus activity coefficient (PAC) ranged from 1.5 to 13.8% within 0–100 cm soil depth. The largest PAC value was observed under OM treatment at 0–40 cm soil depth and under NPK + S treatment at 40–100 cm soil depth. The Ca_2_-P and Ca_8_-P concentrations increased significantly by 0.5–7.5 times and 0.5–10.4 times, respectively in OM treatment with the largest value in 0–40 cm soil depth over CK treatment. The Al-P concentration under NPK + S and OM treatments increased throughout the soil profile. The OM treatment increased all Po concentrations in the 0–40 cm soil depth, while NPK and NPK + S treatments increased labile organic P, moderately labile organic P, and highly stable organic P in the 0–20 cm soil depth. Thus, the application of fertilizer and straw, or organic manure may enhance inorganic and organic P pool in a Mollisol in Northeast China. Thus, organic manure application in the subsoil as a potential P source and their impact should be considered in developing management practices and policies regarding nutrient management.

## Introduction

Phosphorous (P) is an essential major nutrient for plant development, growth, and production. Maintenance of adequate amounts of soil P through application of inorganic and/or organic P is critical for the long-term sustainability of our limited land-based cropping systems^1^. However, continuous, and/or long-term application of P may accumulate in soil and its bio-available form may change. On the other hand, P may be depleted in soil to cause P deficiencies in crops if the outputs exceed inputs in the long term. However, about 71% of the global farmlands are in surplus of P, indicating an unbalance in the P distribution^2^. The amount of P fertilizer application greater than the crops’ demand contribute to the soil P pool and increase its accumulation in soil^3,4^. In addition, often low P use efficiency contribute to the P accumulation in soil. Plant-available soil P status in China is characterized as surplus due to over application and low P use efficiency^5,6^. Song et al.^7^ have reported that the continuous application of chemical fertilizers alone and in combination with animal manures clearly contributed to a P surplus in Mollisol in Northeast China. However, how different fractions of P contribute to this surplus is rather poorly understood and documented.

Phosphorus is present in soil as part of the inorganic (e.g. Fe-P, Ca_2_-P, Ca_8_-P and Al-P) and organic molecules and is classified as inorganic and organic fractions of P, respectively. Similarly, based on the availability, P in soil is often classified into different pools including water soluble, labile, and non-labile. These fractions and pools of P may exist in different amounts and proportions, depending on the soil type and management practices. For example, Song et al.^7^ reported presence of organic P in Mollisol from Northeast China as high as 42–69% of the total P present in soil due to high amount of soil organic matter. One fraction of P can also be transformed to another under certain conditions^8^. Characteristics of these fractions and their dynamics or changes from one to another have been studied in many soils around the world^9–11^. For example, application of P fertilizer can replenish the labile P pool and maintain or increase soil fertility. The sink of P fertilizer in soil can be in the form of labile pool^12^ or even in some more stable pools such as acid-extractable or residual pool of P^13–15^. Phosphorous from water soluble P fertilizer can be transformed quickly to inorganic P including Fe-P, Ca_2_-P, Ca_8_-P and Al-P in calcareous soil^16^.

Thus, fertilizer application is one of the important factors dictating the abundance of different P fractions and pools of P. Without P addition, P pool is mainly considered as non-labile but may become increasingly available to plants at variable amounts depending on soil type and other environmental conditions^17,18^. Long-term application of animal manures and other organic amendments have shown to increase soil total, available, and soluble P concentrations and at soil depths^19–21^. Whalen and Chang^22^ have reported that total and available P pools increased greatly in soils amended with manure annually for 16 years and were 1.2–3.8 and 0.8–1.9 Mg P ha^−1^ greater than in the soils that did not receive any manure. Similarly, P in animal manure and straw can contribute differently towards soil P than P in chemical fertilizer. When the organic amendments are incorporated into soil, the P is influenced by microbial and enzyme activities, composition of the amendment, and the rhizosphere processes^23^. Unlike chemical fertilizer, organic amendment can provide more plant-available P^24^ through organic P mineralization. During the decomposition of organic amendment, a small amount of low-soluble P can also be dissolved in water. However, these processes are dominant at different depths and different parts of the soil profile.

Until now, the majority of soil P research has focused on the topsoil. Little attention has given to the whole soil profile (0–100 cm) which may act as an additional source/sink for plant available P. Additionally, P distribution or status at depths may also be impacted by long-term fertilization. In the northeast grain region of China where water is one of the important factors to successful grain production^25^, the contribution of subsoil P needs to be determined considering that the root of crops may penetrate deep into the soil profile, especially later in the growing season^26^. Wang et al.^26^ suggested that crops accessed significant amounts of P from 10–30 cm of soil depth irrespective of P fertilizer application. However, the subsoil P should be considered critically to improve the soil P status and long-term sustainability of the system. Similarly, the long-term application of different sources of P can have a major influence on different fractions and pools of P at depths and the information is critical to the sustainable development of the cropping system in the black soil (Mollisol) regions of China and elsewhere with similar weather and soils.

Thus, the objectives of this study were to (1) determine the effect of long-term (after 12-year) application of chemical fertilizer, organic amendment and straw on P fractions and pools in black soil from Northeast China, and (2) quantify soil P variability at depths as influenced by agricultural practices.

## Materials and methods

### Study site

The experimental site is located at the State Key Experimental Station of Agroecology of the Chinese Academy of Sciences in Hailun county, Heilongjiang Province (47° 26′ N, 126° 38′ E) (Hailun Station). The station is location at the center of Mollisol region in northeast China. The climate is typical temperate continental monsoon with mean annual temperature of 1.5 °C. The mean annual rainfall is 500–600 mm in this region and the annual accumulated temperature (≥ 10 °C) is 2,450–2,500 °C. The annual sunshine duration is 2,600–2,800 h and the frost-free period is about 120 days. The soil of this region is classified as Mollisol or fine-loamy, mixed, Udic Haploborolls according to the USDA soil Taxonomy. The soil is mainly derived from the sedimentary parent materials. Maize (*Zea mays* L.) is the major crop in this region, which are generally planted in May and harvested in October. The experimental site was a native prairie before the start of crop cultivation about 200 years ago. No fertilizer was applied until crop system was established about 160 years ago. Farm manure was applied as a fertilizer for about 20 years followed by chemical fertilizers (NPK) for another 20 years (from the history of the farm) before a long-term experiment was setup in 2003. At some experimental plots, NPK fertilizers were applied and incorporated.

### Experimental design

A long-term experiment was established at the Hailun station in May 2003. Twelve experimental plots of 21 m^2^ area (4.2 m wide by 5.0 m long) separated by 0.7 m cement barriers were set in completely randomized design with four treatments and four replications. Continuous maize is the main crop in this long-term experiment representing regional practices. The four treatments included application of (1) nitrogen, phosphorus, and potassium fertilizers (NPK), (2) NPK fertilizers plus maize straw incorporation (NPK + S), (3) pig manure (OM) and (4) no fertilizer (CK). Urea, diammonium phosphate and potassium sulfate were applied at the rate of 112.5 kg N, 60 kg P_2_O_5_, and 45 kg K_2_O ha^−1^ year^−1^. All maize straw in corresponding NPK + S treatment plots were incorporated into the soil following recent practice changes. Recently, Chinese government is encouraging farmers to directly incorporate all straw into the soil, which can effectively control various pollutants emission derived from directly straw burning in the field^27^. Pig manure at 15,000 kg ha^−1^, commonly adopted in this area with the aim of enhancing soil fertility^7^, was incorporated into the top 0–20 cm soil layers in the OM treatment. The maize straw contained average total P of 0.7 g kg^−1^, the pig manure contained average total N, P and K concentrations of 22.1, 2.6 and 2.4 g kg^−1^, respectively, and an average organic P of 2.3 g kg^−1^. Baseline soil samples for selective soil physical and chemical properties prior to establishing treatments were collected in 2003 and analyzed in laboratory (Table 1).

**Table 1:** Physical and chemical properties of the initial soils sampled in 2003.

Soil depth (cm)	%			Soil organic carbon (g/kg)	Available phosphorus (mg/kg)	Total phosphorus (g/kg)	Bulk density (g/cm^3^)	pH
	Sand > 0.02 mm	Silt 0.002–0.02 mm	Clay < 0.002 mm					
0–10	36.71	33.08	30.21	22.81	28.07	0.81	1.00	6.54
10–20	36.54	31.27	32.19	21.43	46.61	0.86	1.09	6.59
20–40	32.78	31.66	35.56	20.37	13.28	0.70	1.22	6.61
40–60	30.21	30.81	38.98	13.17	13.50	0.58	1.22	6.69
60–80	28.45	32.14	39.41	10.87	20.17	0.61	1.25	6.78
80–100	27.89	31.90	40.21	8.58	20.26	0.55	1.32	6.54

### Soil sampling

In 2014, all plots were sampled down to 100 cm after 12-year of cultivation. Ten soil cores (3.5 cm diameter) were randomly collected from each experimental plot using a customized soil auger. The soil samples were collected at six depths: 0–10 cm, 10–20 cm, 20–40 cm, 40–60 cm, 60–80 cm, and 80–100 cm. Soil samples were processed in the laboratory by removing any visible plant residues and stones larger than 2 mm immediately after sampling. Soil samples were then air-dried.

### Soil sample analysis

The air-dried soil samples were ground to pass through a 2 mm sieve for laboratory analysis. Soil samples were digested in a tri-acid mixture (HNO_3_, HClO_4_, and H_2_SO_4_ at a 3:1:1 ratio) for determining total phosphorus (Total P). The P concentration in the digest was determined colormeterically using the vanado-molybdate-yellow color method^28^. Soil organic phosphorous (Po) was determined by combustion at 550 °C and extraction with 4 M H_2_SO_4_^29^. Olsen method^30^ was used to determine available P using the colormetric molybdenum method after the extraction with 0.5 mol NaHCO_3_ L^−1^.

### Soil organic phosphorous fraction

**Figure 1:**
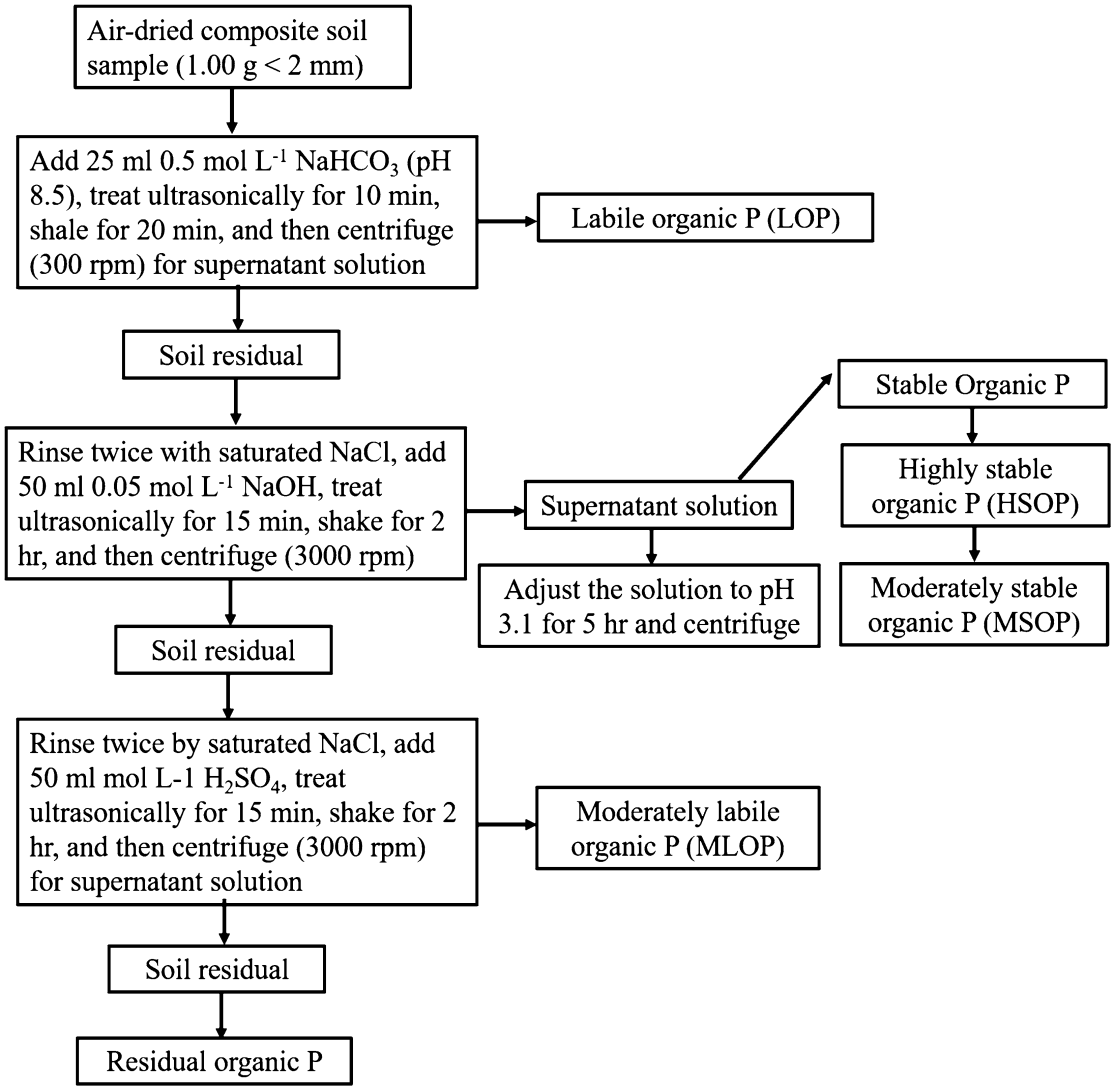
Scheme of sequence extraction of organic P in a Mollisol.

Soil organic phosphorous (Po) fractions were separated into four forms using a modified sequential extraction procedure developed by Bowman and Cole^31^ and modified by Fan et al.^32^. A flow diagram for the soil organic P fractionation procedure is outlined in Fig. 1. Highly stable organic phosphorous (HSOP) was determined from the difference between stable organic P and moderately stable organic P (MSOP). The MSOP and HSOP represented fulvic and humic acid P, respectively. The P concentration in all extracts was determined colorimetrically according to the method by Murphy and Riley^33^.

### Soil inorganic P fraction

Inorganic P (Pi) fractions were measured according to a fractionation scheme of Jiang and Gu^34^, which is based on the method described by Hedley et al.^35^. Briefly, the fractionation involved a sequential extraction with (1) 0.25 M NaHCO_3_ (pH 7.5) to extract Ca_2_-P, (2) 0.5 M CH_3_COONH_4_ (pH 4.2) to extract Ca_8_-P, (3) 0.5 M NH_4_F (pH 8.2) to remove Al-P, (4) 0.1 M NaOH-0.1 M Na_2_CO_3_ to obtain Fe-P, (5) 0.3 M sodium citrate-Na_2_S_2_O_4_-0.5 M NaOH to obtain the occluded P, and (6) 0.5 M H_2_SO_4_ to extract Ca_10_-P. These fractions were designated as NaHCO_3_-soluble P (Ca_2_-P), NH_4_Ac-soluble P (Ca_8_-P), NH_4_F-soluble P (Al-P), NaOH-NaCO_3_-soluble P (Fe-P), occluded P (O-P), and H_2_SO_4_-soluble P (Ca_10_-P), respectively.

### Calculation and statistical analysis

The soil total P (TP), available P (AP) and P fractions accumulations in soil layers of the treatments were determined using Eqs. (1) and (2)^36^:

1 $$ {\text{Soil}}\;{\text{accumulation}}\;\left( {{\text{kg ha}}^{{ - {1}}} } \right) = {\text{the soil TP concentrations }}\left( {{\text{g kg}}^{{ - {1}}} } \right) \times {\text{the bulk density }}\left( {{\text{g cm}}^{{ - {3}}} } \right) \times {\text{ soil depth }}\left( {{\text{cm}}} \right) \times { 1}00 $$

2 $$ {\text{Soil}}\;{\text{accumulation}}\;\left( {{\text{kg ha}}^{{ - {1}}} } \right) = {\text{ the soil AP and P concentrations }}\left( {{\text{mg kg}}^{{ - {1}}} } \right) \times {\text{ the bulk density }}\left( {{\text{g cm}}^{{ - {3}}} } \right) \times {\text{soil depth }}\left( {{\text{cm}}} \right)/{1}0 $$

The experiment was performed as a 4-factorial experiment in a completely randomized design, with four replicates. Analysis of variance (ANOVA) for evaluating the effects of chemical fertilizer, organic manure and straw incorporation treatments on soil total P, available P, and P fractions was carried out in SPSS statistical software (SPSS, 1998). Standard errors were calculated for mean values of determinations. The multiple comparison test for mean separation was completed using Fisher’s (protected) LSD at a 0.05 significance level.

## Results

### Total P and available P

**Figure 2:**
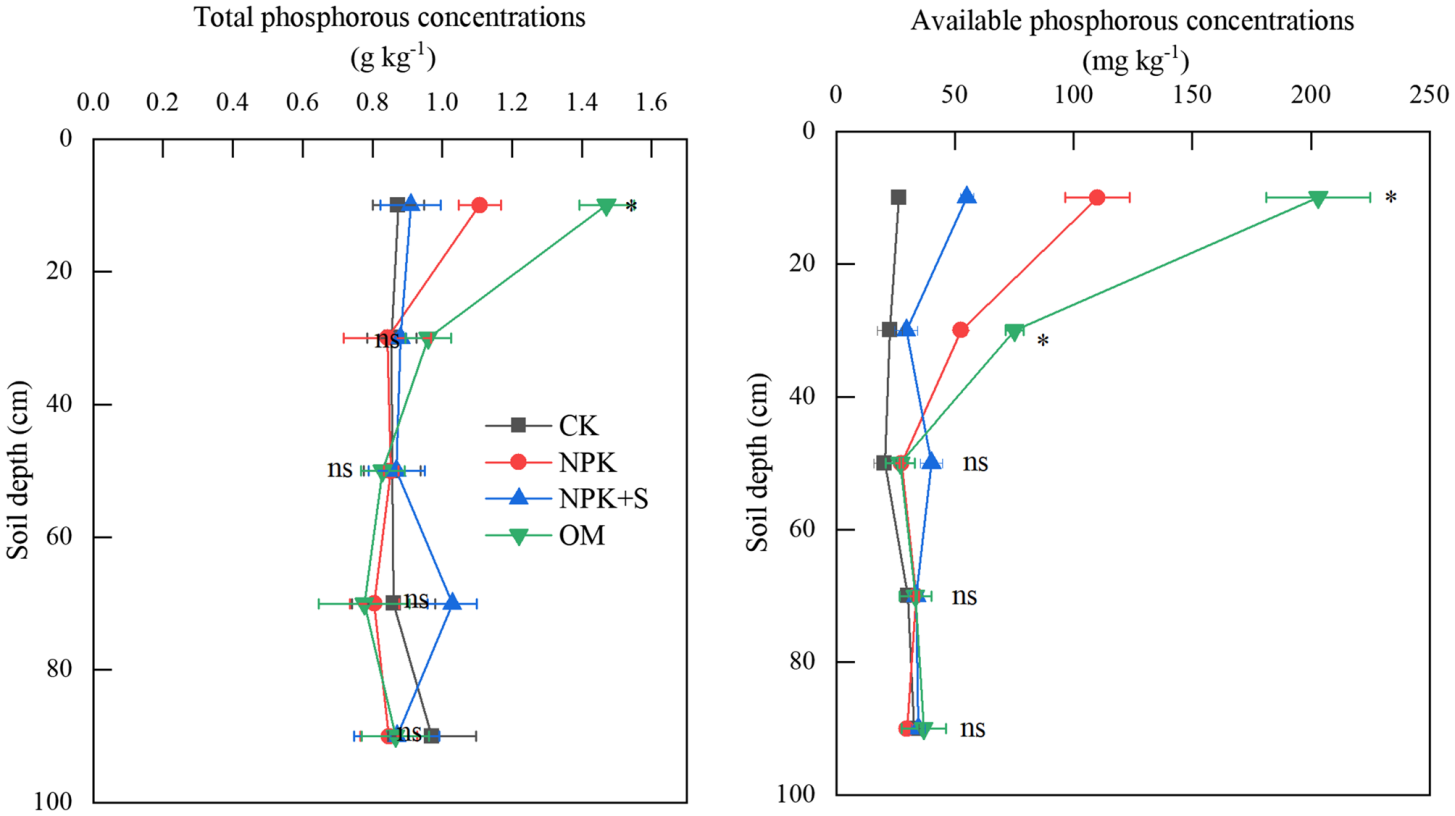
Effect of long-term application of chemical fertilizer, organic manure, and straw on total phosphorus (P) and available P concentrations. * indicates significant difference at *P* < 0.05 level at same soil depth, ns indicates no significant difference between the total phosphorus (P) and available P concentrations at the same soil depth. CK, no fertilizer application; NPK, chemical fertilizer application; NPK + S, chemical fertilizer plus maize straw incorporation; OM, organic manure application.

Fertilizer application significantly (*P* < 0.05) increased total P concentration within 0–20 cm soil depth and available P concentration within 0–40 cm soil depth (Fig. 2). Compared to CK, total P concentration in the 0–20 cm soil depth was increased by 26.6%, 4.1%, and 68.2% in NPK, NPK + S, and OM treatments, respectively, while available P concentration was increased by 315.9%, 108.3% and 667.5% in NPK, NPKS and OM treatments, respectively. Phosphorous was accumulated in the 0–40 cm soil depth in both NPK and OM treatments, majority of which was in the cultivated 0–20 cm soil depth (Fig. 2). The majority of P was accumulated in the 60–80 cm soil depth under NPK + S treatment, but no significant difference was observed among treatments (*P* > 0.05). The highest increase in available P concentration in NPK + S treatment observed in the 60–100 cm soil depth, with the increase of 111% and 115% in 60–80 cm and 80–100 cm soil depths, respectively, over CK treatment.

### Phosphorous activity constant

**Figure 3:**
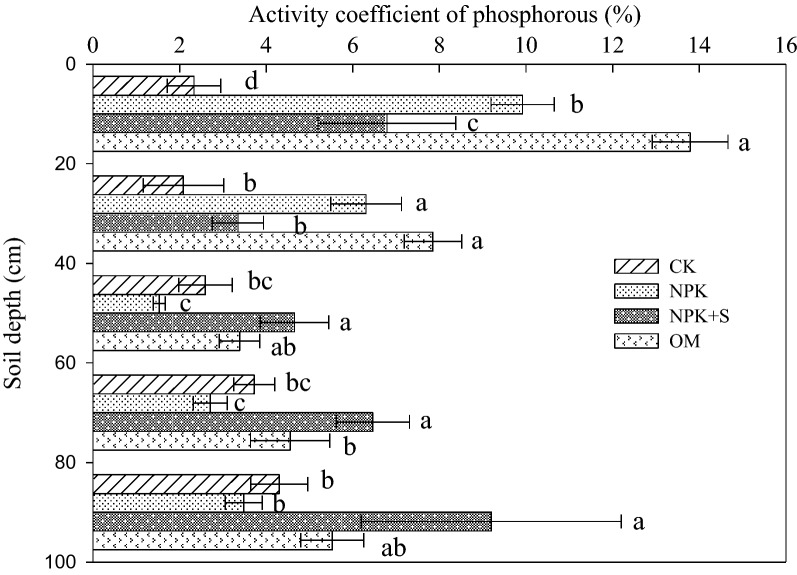
Effect of long-term fertilization on phosphorous activation constant in Mollisol profiles. Different letters in the bars of same depth indicates significant difference at *P* < 0.05 level among different treatments. CK, no fertilizer application; NPK, chemical fertilizer application; NPK + S, chemical fertilizer plus maize straw incorporation; OM, organic manure application.

The phosphorus activity coefficient (PAC, the ratio of available P to total P) is an important indicator of soil P availability and the transformation of P fractions^37^. When the PAC is less than 2.0%, the total P is not easily converted to available P^38^. The PAC ranged from 1.5% to 13.8% within 0–100 cm soil depth and the largest value (> 3.6%) was associated with OM treatment, especially at the 0–20 and 20–40 cm soil depths (Fig. 3). The PAC values under NPK, NPK + S and OM treatments increased by 7.6%, 4.5% and 11.5% in the 0–20 cm soil depth and 4.2%, 1.3%, and 5.8% in 20–40 cm soil depth, respectively as compared to the CK treatment. However, PAC value for soil depth below 40 cm showed the trend, NPK < CK < OM < NPK + S treatments (*P* < 0.05).

### The accumulation of total P and available P in soil profile

Total P accumulation under NPK, NPK + S and OM treatments within the 0–100 cm soil profile was 511, 1,068 and 1,353 kg P ha^−1^ more than that under CK treatment (Table 2). Increase in total P in NPK and OM treatments within the top 0–20 cm soil depth accounted for 95.9% and 79.5% of total P increase within 0–100 cm soil profile, respectively. However, total P in NPK + S treatment was mainly accumulated between 20–100 cm soil depths, with increased values of 215, 201, 556 and 90 kg P ha^−1^ for 20–40, 40–60, 60–80 and 80–100 cm soil depths, respectively over the CK treatment (Table 2). The NPK and OM treatments showed increase in available P concentration at the 0–40 cm soil depths (*P* < 0.05). The available P concentrations under NPK and OM treatments increased by 181.7 and 356.1 kg P ha^−1^ in the 0–20 cm soil depths, and 69.7 and 127.0 kg P ha^−1^ for 20–40 cm soil depth compared to that under CK treatment. Available P under NPK + S treatment increased throughout the entire 0–100 cm soil profile compared to that associated with the CK treatment (*P* < 0.05) (Table 2).

**Table 2:** The effect of different treatments on total phosphorus (P) and available P accumulation in soil profiles (kg ha^−1^).

Soil depth	Total P				Available P			
(cm)	CK	NPK	NPK + S	OM	CK	NPK	NPK + S	OM
0–20	1923c	2413b	1929c	2999a	58.21d	239.94b	114.62c	414.29a
20–40	1898b	2122b	2113a	2263ab	50.11d	119.22b	68.37c	177.10a
40–60	2157a	2151a	2358a	2190a	51.56b	69.04ab	98.62a	68.12b
60–80	2376ab	2222b	2932a	2344ab	83.37a	92.70a	93.00a	92.06a
80–100	2497a	2454a	2587a	2407a	91.22a	82.70a	95.98a	102.47a
Total	10850a	11361a	11918a	12204a	334.47d	604.14b	470.58c	854.03a

CK, no fertilizer application, NPK: chemical fertilizer application, NPK + S: chemical fertilizer plus maize straw incorporation, OM: organic manure application.

Values followed by different letters at the same row differ significantly at *P* < 0.05.

### P fractions

**Figure 4:**
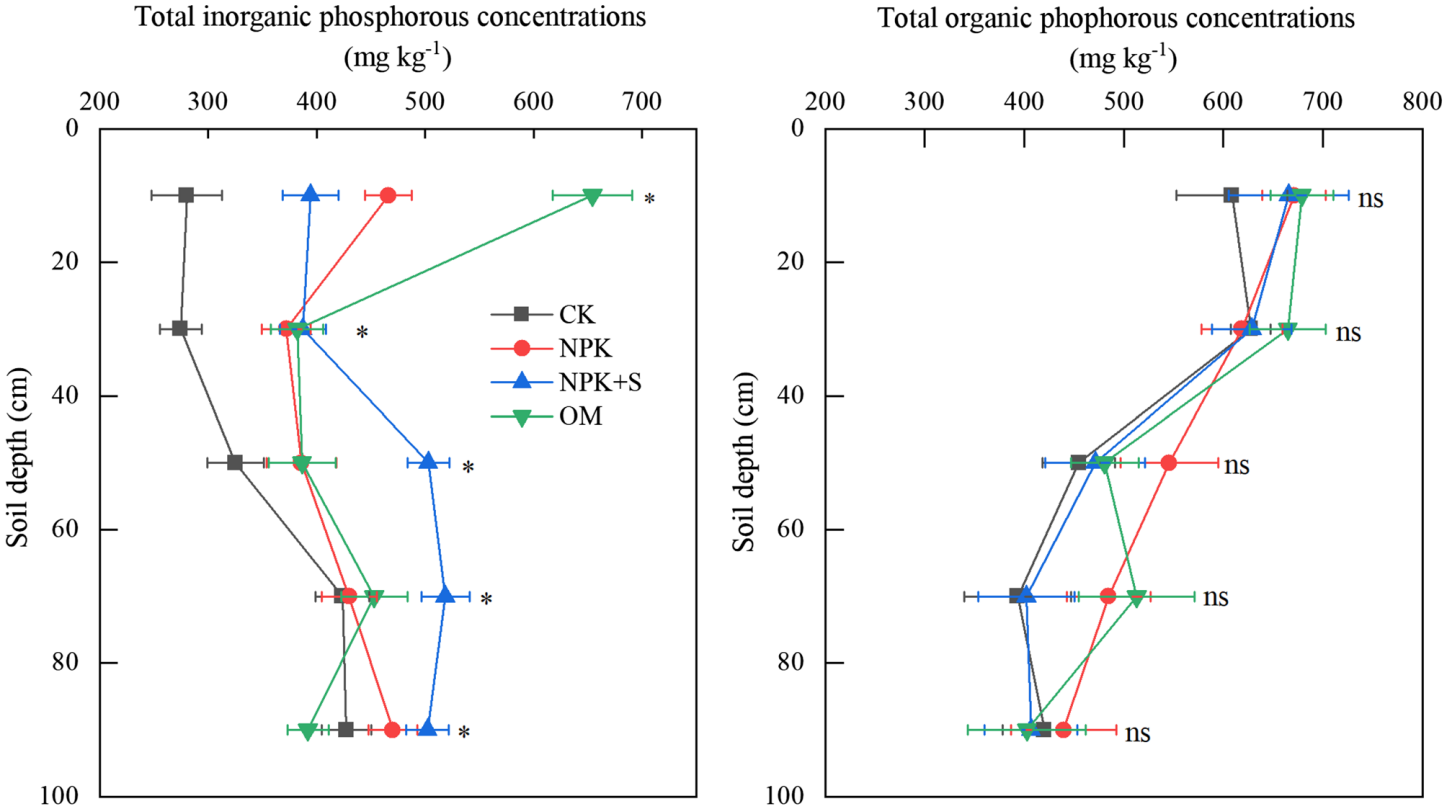
Effect of long-term application of chemical fertilizer, organic manure and straw on the distribution of organic-P and inorganic-P contents. * indicates significant difference at *P* < 0.05 level at the same soil depth, ns indicates no significant difference regarding the total inorganic and organic P concentrations at the same soil depth. CK, no fertilizer application; NPK, chemical fertilizer application; NPK + S, chemical fertilizer plus maize straw incorporation; OM, organic manure application.

Organic P (Po) concentration decreased with the increase of soil depth regardless of treatment, while inorganic P (Pi) concentration increased under CK and NPK + S treatments and decreased under NPK and OM treatments (Fig. 4). The P fertilizer application significantly increased Pi concentration in the 0–100 cm soil depth (*P* < 0.05) and Po concentration at 40–80 cm soil depth (*P* < 0.05) compared with that of the CK treatment. The Pi under NPK and OM treatments accumulated mainly in the 0–20 cm soil depth resulting in higher Pi concentration of 12.4% and 62.1%, respectively, than that at deeper soil depths (Fig. 4). Applied P fertilizer mainly presented as Pi in 0–40 cm soil depth for the NPK and OM treatments, and in 0–100 cm soil depth for NPK + S treatment over CK treatment.

**Figure 5:**
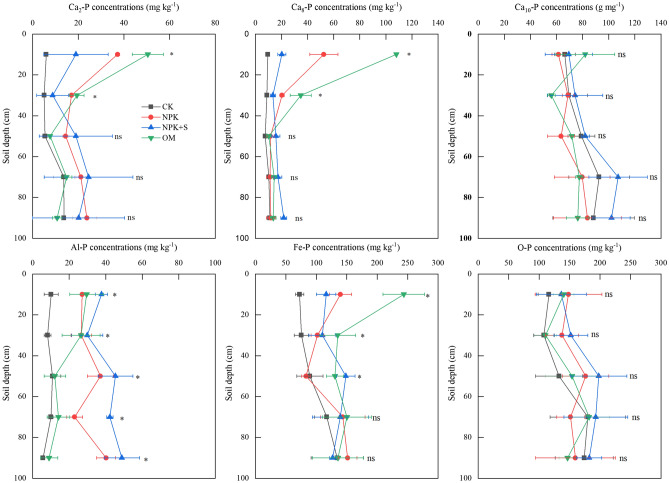
Effect of long-term application of chemical fertilizer, organic manure and straw on the distribution of inorganic P fractions. * indicates significant difference at *P* < 0.05 level at the same soil depth, ns indicates no significant difference regarding the Ca_2_-P, Ca_8_-P, Al-P, Fe-P, O-P and Ca_10_-P concentrations at the same soil depth. CK, no fertilizer application; NPK, chemical fertilizer application; NPK + S, chemical fertilizer plus maize straw incorporation; OM, organic manure application.

In general, concentration of all Pi fraction under NPK + S, NPK and OM treatments increased depending on soil depth except for Ca_10_-P compared to that associated with the CK treatment (Fig. 5). Different fertilizer treatments have significantly impacted Ca_2_-P and Ca_8_-P concentrations as deep as 0–40 cm depth (*P* < 0.05), showing a decreasing trend as OM > NPK > NPK + S. Compared with CK treatment, the Ca_2_-P concentrations associated with OM, NPK and NPK + S treatments showed significant increase of 5.2 times, 7.5 times and 1.4 times, respectively in the 0–20 cm soil depth and 2.4 times, 2.8 times and 0.5 times, respectively in 20–40 cm soil depth (*P* < 0.05). The Ca_8_-P concentration associated with NPK, OM and NPK + S treatments showed significant increase of 4.5 times, 10.4 times and 1.1 times, respectively in the 0–20 cm soil depth and 1.3 times, 2.9 times and 0.5 times, respectively in the 20–40 cm soil depth (*P* < 0.05). The NPK and NPK + S treatments showed significant increase in Al-P concentrations of 1.30 times and 7.61 times, respectively within 0–100 cm soil depth (*P* < 0.05), while OM treatment showed significant increase of Fe-P concentration of 79.0–235.8% within 0–60 cm soil depths (*P* < 0.05), compared with the CK treatment. Fertilizer application increased O-P concentration with significant differences observed between different treatments (*P* < 0.05).

**Figure 6:**
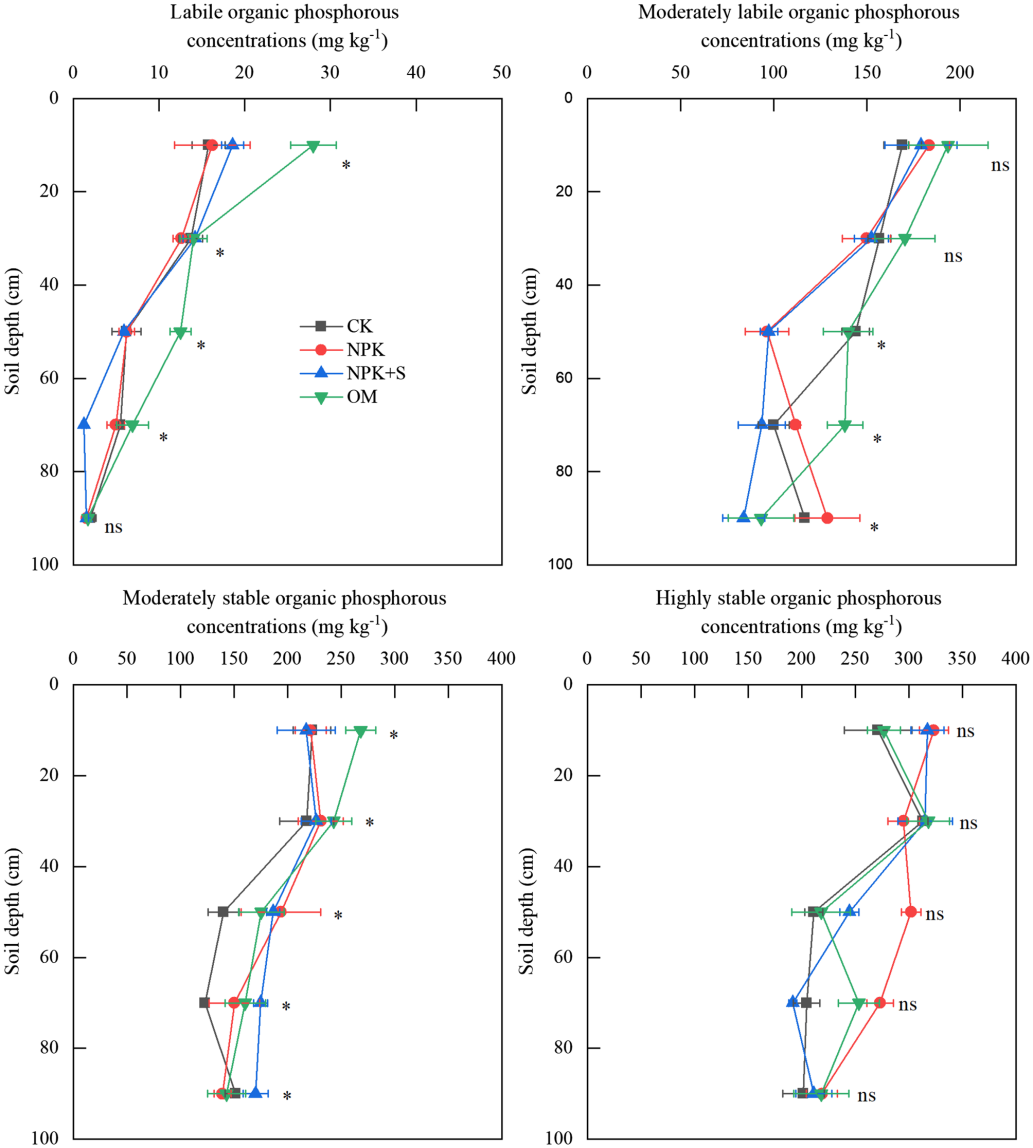
Effect of long-term application of chemical fertilizer, organic manure and straw on the distribution of organic P fractions. * indicates significant difference at *P* < 0.05 level at the same soil depth, ns indicates no significant difference regarding labile organic P, moderately labile organic P, moderately stable organic P and highly stable organic P concentrations at the same soil depth. CK, no fertilizer application; NPK, chemical fertilizer application; NPK + S, chemical fertilizer plus maize straw incorporation; OM, organic manure application.

The concentrations of different Po fractions decreased with the increase of soil depth (Fig. 6) except for HSOP. The added P fertilizer mainly transformed to MSOP and HSOP (Fig. 5). The LOP and MLOP forms under OM treatment increased by 77.4% and 14.45%, respectively in the 0–20 cm soil depth as compared to that associated with the CK treatment. The LOP under NPK + S increased by 17.7% and 3.5% for 0–20 cm and 20–40 cm soil depth, respectively, compared with CK treatment. MSOP concentrations under NPK and NPK + S treatments decreased by 0.6% and 2.4% within 0–20 cm soil depth, while increased by 6.0–38.3% and 4.0–12.4% within 20–80 cm soil depths, respectively. OM treatment significantly increased MSOP concentration by 11.6–30.3% within 0–80 cm soil depth with the peak at 60–80 cm soil depth. Generally, P fertilizer application increased HSOP concentration at 0–100 cm soil profile by 8.5–45.0%, 0.7–17.0% and 1.6–23.8% for NPK, NPK + S and OM treatments, respectively, with significantly different at 20–60 cm soil depth.

### Accumulation of different P fractions

Total P accumulation increased mainly in the 0–20 cm soil depth as impacted by long-term fertilization in this study (Table 2). Compared with CK treatment, the accumulation of Fe-P, Ca_8_-P, Ca_2_-P, O-P and Al-P in NPK treatment increased by 144.9, 93.5, 68.0, 67.8, and 36.8 kg P ha^−1^, respectively; while the accumulation of Fe-P, Ca_8_-P, Ca_2_-P, MSOP, and Al-P associated with OM treatment increased by 347.3, 203.8, 91.8, 129.5 and 39.2 kg P ha^−1^, respectively. The accumulation of Fe-P and Al-P associated with NPK + S treatment were increased as well (Table 3).

**Table 3:** The effect of treatments on the accumulation of different P fractions in the 0–20 cm soil depth (kg ha^−1^).

Treatment	LOP	MLOP	MSOP	HSOP	Ca_2_-P	Ca_8_-P	Al-P	Fe-P	O-P	Ca_10_-P
CK	33.24a	372.14b	416.95b	597.01b	13.09c	20.82c	22.28b	159.69c	255.50a	146.13a
NPK	35.36a	399.71b	453.63b	661.35a	81.05b	114.34b	59.03a	304.60b	323.27a	133.97a
OM	49.67a	442.88a	546.43a	667.37a	104.93a	224.62a	61.52a	506.98a	292.23a	170.54a
NPK + S	44.72a	365.29b	443.57b	647.50a	28.78c	41.15c	76.83a	237.13bc	278.66a	141.71a

Values followed by different letters at the same row differ significantly at *P* < 0.05.

CK, no fertilizer application; NPK, chemical fertilizer application; NPK + S, chemical fertilizer plus maize straw incorporation; OM, organic manure application.

LOP, labile organic P; MLOP, moderately labile organic P; MSOP, moderately stable organic P; HSOP, high stable organic P.

### Proportions of P fractions

**Figure 7:**
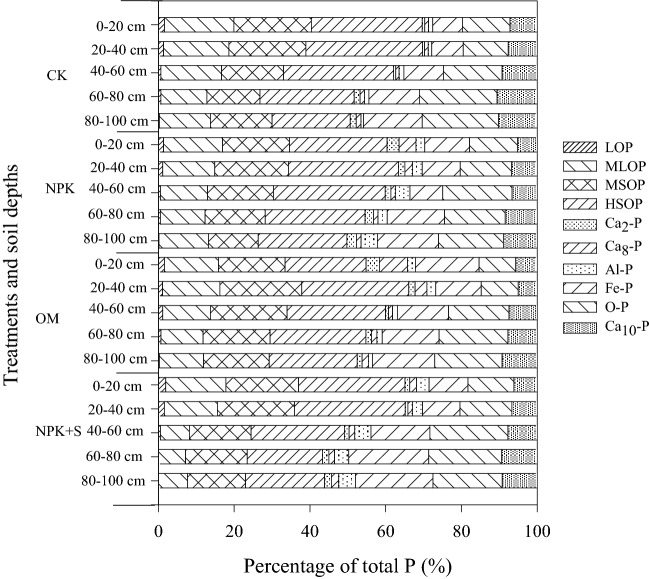
Effect of long-term application of chemical fertilizer, organic manure and straw on the percentage of different P fractions. LOP, labile organic P; MLOP: moderately labile organic P; MSOP: moderately stable organic P; HSOP: highly stable organic P. CK: no fertilizer application, NPK: chemical fertilizer application, NPK + S: chemical fertilizer plus maize straw incorporation, OM: organic manure application.

The concentrations of different P fractions increased significantly in the 0–40 cm soil depth (*P* < 0.05) (Fig. 7). Compared with CK, the proportions of Po fractions of the total P associated with NPK and NPK + S treatments decreased by 0.3–3.5%, while the proportions of Ca_2_-P, Ca_8_-P and Fe-P of total P associated under NPK and NPK + S treatments increased by 1.0–4.0 percentage. The greatest variations among these fractions were observed at the 0–20 cm soil depth. However, OM treatment did not impact the proportion of different Po fractions of the total P compared with CK treatment. Field treatments did not impact the proportion of different P fractions of the total P below 40 cm soil depths.

## Discussion

### Effects of chemical fertilizers soil phosphorous and phosphorous fractions

Use of fertilizer as nutrient source is critical for crop production. In P deficient soils, the application of P fertilizer can help increase crop yield. However, crop response to P fertilizer may not be significant when available P concentration in soil is higher than an optimum or agronomic threshold value^39^. Tang et al.^40^ documented average agronomic threshold values for maize and wheat to be 15.3 mg P kg^−1^ soil and 16.3 mg P kg^−1^ soil, respectively in Calcareous soils in China. The threshold values of available P for maize and wheat in Haplic Luvisol in northern China was reported to be about 12.5 mg P kg^−1^ soil^41^. Soil P concentration under CK treatment depleted after continuous maize grown for 12 years in this study. The amount of total P removed from the soil was as high as 302.4 kg P ha^−1^ soil as estimated based on the annual biomass, grain yield, and total P concentration of both^42^. However, the available P concentration at CK treatment was as high as 19.8 mg P kg^−1^ soil in the 0–20 cm soil depth (Fig. 2), which was higher than the agronomic threshold value of available P for most soils across China^26,40,41^. However, these values were still lower than that in the lower soil depths, indicating an abundance of soil P in Mollisol. But P fertilizer application is still necessary in Mollisol in Northeast China due to low soil temperature causing low P availability to crops early in the growing season^6^. In this study, P fertilizer was applied at the rate of 45 kg P_2_O_5_ ha^−1^ soil (NPK treatment) and was lower than the removal rate of P from soil^42^. This may have contributed to the P deficiency of 259.2 kg P ha^−1^ soil estimated based on the annual biomass, grain yield, and total P concentration of both^42^. However, available P concentration was as high as 110.1 mg P kg^−1^ soil in the 0–20 cm depth. The P removal rate under NPK + S treatment was approximately equal to the P fertilizer input. The available P concentration in the 0–20 cm soil depth under NPK + S treatment was 55.1 mg P kg^−1^ soil and was less than that under the NPK treatment. The total amount of P fertilizer added under the OM treatment was 964.8 kg P_2_O_5_ ha^−1^ over 12 years, which exceeded the amount of removed P over the same period. This excess amount of available P was reflected in the soil test of P of 203.1 mg P kg^−1^ soil.

In this study, the main P fractions accumulated with no P fertilizer application were Ca_2_-P, Ca_8_-P, Al-P and Fe-P (Fig. 6). Han et al.^43^ documented that the initial concentration of Fe-P, Al-P and Ca-P correlated significantly (*P* < 0.05) with crop biomass in Albic Luvisols. Availability of different P fractions for plant P uptake varied within P pools. The Ca_2_-P can be readily available to plant, which represents monocalcium phosphates and dicalcium phosphate equivalents. The Ca_8_-P can be partly available to plants, which represents a group of phosphates with chemical structure similar to Ca_8_H_2_(PO_4_)6·nH_2_O. The Al-P and Fe-P have low availability to plants which represent aluminum phosphates and iron phosphates, respectively^34^. Therefore, the forms of Ca_2_-P, Ca_8_-P, Al-P and Fe-P can be absorbed first where no P fertilizer was applied^7,44^. This means that when P is not supplied, the crop plants are able to access these sparingly soluble P fractions from soil. Alternatively, the plant uptake resulted in a substantial decrease in available P within 0–60 cm soil profile (Fig. 2), which would enhance dissolution of the sparingly soluble P. The concentrations of MSOP and HSOP were decreased under treatment with no fertilizer (Table 3). This indicated that moderately stable Po and high stable Po may change to labile Po and Pi possibly due to the long-term tillage and root activity^14^.

The P applied through chemical fertilizer existed in the 0–20 cm soil depth in the form of Fe-P, Ca_8_-P and Ca_2_-P (Table 3) and increased the concentrations of Ca_2_-P, Ca_8_-P, Al-P and Fe-P significantly (*P* < 0.05) (Fig. 6). However, there was no significant impact on Po concentration (Fig. 7). This finding is consistent with Li et al.^5^ who demonstrated that long-term single chemical P fertilizer application increased almost all forms of Pi, but only marginally the Po fraction. The concentration of available P under NPK treatment in 0–20 cm soil depth was significantly greater than that in the CK treatment (Fig. 2). The increase in available P accumulation accounted for 62.5% of total P accumulation (Table 2). This may be explained by the relatively high Pi availability that was associated with applied fertilizer (Fig. 2). Application of P fertilizer can replenish the labile P pools and maintain or increase soil fertility^5,42^. The PAC values of NPK treatment was higher than that in the CK treatment (Fig. 3). It was reported after an incubation experiment that the transformation of different P fractions occurred after 156-day application of sludge. During this time, the P transformed to Pi for plant uptake and different P fractions became stable^45^. Plant P uptake could result in different P fractions continuously transforming in soil.

### Effects of organic amendment soil phosphorous and phosphorous fractions

Over ninety percent of total P associated with OM treatment accumulated in the 0–20 cm soil depth. This led to increase in the concentration of Pi (203.1 mg P kg^−1^ soil) over Po, enhancing P availability by 66.3% of the total P accumulation in soil under OM treatment. Organic manure can indeed enhance soil P availability by adding P in soil after mineralization or chelating with P fixating compounds including iron, calcium, and aluminum^46^. Taka-hashi^47^ reported that manure initially containing some amounts of Ca_2_-P and Fe-P fractions may contribute to the increase of Ca_2_-P and Fe-P fractions. Long-term application of organic amendment increased accumulation of Fe-P, Ca_8_-P, Ca_2_-P and MSOP in the soil profile (Table 3). This may be attributed to the potential of organic P in manure that effectively mineralized by microorganisms and thus transferred into various inorganic P fractions^7^. The Po in soil under pasture with long-term (> 20 year) organic manure application was mineralized easily, leading to increase in the P pool, which could be responsible for the fact that phosphodiester was higher than that in cropping system in Canada, could be mineralized easily^48^. The accumulation of total and available P under OM treatment increased by 1,354 and 517.83 kg P ha^−1^ within 0–100 cm soil profiles, respectively, compared with CK treatment (Table 2). The concentrations of both Pi and Po under OM treatment increased significantly, compared with CK treatment, which did not significantly change the ratios of Pi and Po to the total P (Fig. 7). This implied that the application of pig manure alone could meet the P demand for maize in the study site. Previous studies have found that higher available P concentration was observed in the treatment with higher rate of manure application^49^, which could be attributed to the possible production of low molecular weight organic acids through microbial decomposition of manure^50^. Zhang et al.^51^ reported that the concentrations of total and available P have shown an increasing trend with increasing manure application rate. However, the amount of organic manure application in agricultural systems should also considered soil P depletion. Because large amount application of organic manure could result in excess P accumulation in soils, which causes a potential risk of nonpoint source pollution^52^.

Straw incorporation could increase the concentrations of phosphomonoester and phosphodiester but may decrease the concentration of available P in soil^53^. In this study, the long-term application of straw (NPK + S) increased the concentrations of Po and Pi in the 0–100 cm soil profile compared to the CK treatment. The treatment with straw incorporation increased the accumulation of total and available P by 1,068 and 136.11 kg P ha^−1^ within 0–100 cm soil profiles, respectively, compared with CK treatment (Table 2). It was interesting that the concentrations of total and available P associated with NPK + S treatment decreased in the 0–20 cm soil depth. This phenomenon was consistent with previous studies which documented that the incorporation of maize straw could result in the decrease of soil available P by increasing microbial immobilization^54^. Wei et al.^55^ also reported that the amount of available P uptake by crops was higher under NPKS treatment than that under NPK treatment. However, the concentrations of Al-P, O-P and Ca_10_-P increased within the 40–100 cm soil depth. Although Han et al.^43^ reported that the vertical movement of P was minimal in this soil type, a substantial amount P was observed in the deep soil under NPK + S treatment. This may be attributed to the large variation in soil water content in the 0–20 cm soil depth under NPK + S treatment, inducing deeper crop root and impacting the transformation of P fractions. But further studies are warranted to determine the mechanisms of P movement in soil profile after straw incorporation.

## Conclusion

Long-term application of P as chemical fertilizer, organic manure, and straw increased total P concentration in the 0–20 cm soil depth and available P concentration in the 0–60 cm soil depth. The accumulation of total and available P in the 0–100 cm soil profile was affected by different fertilization treatments. Long-term P fertilization increased majority of P fractions and PAC values. Generally, the organic manure application increased all Po and Pi fractions’ concentrations in the 0–80 cm soil depth. This suggested that organic manure can increase P pool in the subsoil as potential P source for crop uptake. The straw amendment under the NPK + S treatment contributed to the increase of Pi concentration in the 40–100 cm soil profile. Furthermore, attention should be paid to potential deep soil P movement in order to avoid potential environmental problems. Further studies are warranted to determine the mechanisms of P movement in soil profile.
